# Eco‐Friendly Colloidal Quantum Dot‐Based Luminescent Solar Concentrators

**DOI:** 10.1002/advs.201801967

**Published:** 2019-03-01

**Authors:** Yimin You, Xin Tong, Wenhao Wang, Jiachen Sun, Peng Yu, Haining Ji, Xiaobin Niu, Zhiming M. Wang

**Affiliations:** ^1^ Institute of Fundamental and Frontier Sciences University of Electronic Science and Technology of China Chengdu 610054 P. R. China; ^2^ School of Materials and Energy State Key Laboratory of Electronic Thin Film and Integrated Devices University of Electronic Science and Technology of China Chengdu 610054 P. R. China

**Keywords:** colloidal quantum dots, eco‐friendly, luminescent solar concentrators, optical properties

## Abstract

Luminescent solar concentrators (LSCs) have attracted significant attention as promising solar energy conversion devices for building integrated photovoltaic (PV) systems due to their simple architecture and cost‐effective fabrication. Conventional LSCs are generally comprised of an optical waveguide slab with embedded emissive species and coupled PV cells. Colloidal semiconductor quantum dots (QDs) have been demonstrated as efficient emissive species for high‐performance LSCs because of their outstanding optical properties including tunable absorption and emission spectra covering the ultraviolet/visible to near‐infrared region, high photoluminescence quantum yield, large absorption cross sections, and considerable photostability. However, current commonly used QDs for high‐performance LSCs consist of highly toxic heavy metals (i.e., cadmium and lead), which are fatal to human health and the environment. In this regard, it is highly desired that heavy metal‐free and environmentally friendly QD‐based LSCs are comprehensively studied. Here, notable advances and developments of LSCs based on unary, binary, and ternary eco‐friendly QDs are presented. The synthetic approaches, optical properties of these eco‐friendly QDs, and consequent device performance of QD‐based LSCs are discussed in detail. A brief outlook pointing out the existing challenges and prospective developments of eco‐friendly QD‐based LSCs is provided, offering guidelines for future device optimizations and commercialization.

## 1 Introduction

Solar energy is an ideal candidate for replacing traditional fossil fuel due to its environmental‐friendliness and abundance.^1^ Cost‐effective, long‐term stable and high‐efficiency photovoltaic (PV) technologies can directly convert the solar radiation into electrical power, which are promising to address the increasing demand for sustainable and renewable energy.^2^ Over the last several decades, silicon (Si) solar cells have been widely employed in daily life owing to their broad light absorption that matches well with the solar spectrum.^3^ Si solar cells can achieve a very high power conversion efficiency (PCE) up to ≈25% and simultaneously maintain 85% of this original PCE after long‐term operation (≈20 to 30 years), while the cost of Si solar cells is still comparatively high.[[qv: 3a,d,4]] In this regard, a large number of novel PV materials and devices have been recently investigated, examples include various kinds of semiconductor nanomaterials and relevant PV devices such as organic polymer solar cells,^5^ quantum dots (QDs) solar cells,[[qv: 2a,6]] and perovskite solar cells etc.^7^ However, there are still some limitations of these PV materials and devices, for instance, these PV materials and cells can't achieve rather high efficiencies as compared to the Si solar cells, for example, the record PCE of QD‐based solar cell is ≈12%.^8^ Although the perovskite solar cells have obtained comparable PCE (over ≈20%) with respect to Si solar cells, the poor stability still impede their industrial applications.^9^

Luminescent solar concentrators (LSCs) would be the promising devices to optimize the traditional PV systems.^10^ Weber and Lambe have proposed the concept of LSCs in 1976, where a planar solar collector device was fabricated with a luminescent medium that enables the absorption of solar radiation and subsequent collection via total internal reflection (TIR), aiming to improve the efficiency of coupled PV cells by concentrating the incident light on the device.^11^ The idea of LSCs is based on exploiting solar radiation by the use of large‐area devices with a minimal PV material consumption.[[qv: 10a,12]] Combining the silicon‐based PV devices with LSCs can reduce the effective area used to generate identical amount of electricity so as to lessen the device cost.[[qv: 2c]] As the coupled PV cells of LSCs can convert the concentrated solar radiation into electricity so that LSCs enable the fantastic idea of cost‐saving net‐zero buildings.^13^ Organic fluorescent dyes with high quantum yield (QY) are the most commonly used luminescent materials in early studies of LSCs. However, these organic fluorescent dyes for LSCs (e.g., Rhodamine) generally show a narrow absorption spectrum mainly in the ultraviolet (UV) and visible region, thus limiting the light harvesting of the solar spectrum and leading to lower efficiency of LSCs. This is due to the difficulty to extend the intrinsic conjugation length of organic dyes for broad light absorption.[[qv: 10b]] Organic fluorescent dyes are also suffering from the small Stokes shift.^14^ Herein, the Stokes shift represents the difference between their absorption excitation peak and emission peak, i.e., their overlap area.[[qv: 10b,14]] Such small Stokes shift results in large re‐absorption and optical losses that deteriorate the performance of LSCs. Furthermore, the organic dyes usually exhibit poor stability as a result of degradation under illumination.[[qv: 12a]] It is still challenging to explore organic dyes simultaneously possessing excellent optical properties and durability for LSC devices.[[qv: 12c]]

Over several years, QDs have attracted tremendous attention for LSCs applications due to their unique size/composition‐dependent optical properties, including tunable Stokes shift, high photoluminescence quantum yield (PLQY) and decent photo/chemical‐stability.^15^ It is noted that QDs with large Stokes shift can partly solve the problem of re‐absorption loss, thus improving the emission efficiency of LSCs. Moreover, QDs have many other advantages for application in LSCs, such as broad spectral absorption, high absorption coefficient and tunable emission spectra from visible to near‐infrared (NIR) region (wavelength over ≈700 nm). In particular, the NIR emission of QDs is favorable for LSCs as the emitted NIR photons can match well with the maximum spectral response of the Si PV cells, thus leading to a higher device efficiency.^16^

QD‐based LSCs have gone through a rapid development in the last several decades. Many kinds of QDs acting as the phosphors in the LSCs have been studied, most of them belong to II–VI and IV–VI QDs such as CdS/Se and PbS/Se QDs.[[qv: 12c,17]] Unfortunately, these QDs generally contain toxic heavy metals (hereafter the heavy metals refer to toxic elements of Cd and Pb, unless supplementarily stated in this paper), which are fatal to environment protection as well as human health. In this perspective, various kinds of eco‐friendly QDs, including carbon QDs, Si QDs, ternary chalcopyrite compound QDs, gold nanoclusters, etc., have emerged and been employed as phosphors for LSCs devices.^18^ Meanwhile, investigations on large‐area LSCs are rising and the size of LSCs tend to be large to suit the size of building's facades.

Although eco‐friendly QDs possess the great potential to realize high performance LSC devices, there are still several major challenges: i) how to enhance QDs' emission efficiency; ii) how to minimize QDs' overlap between absorption and emission spectrum; iii) how to improve photo/chemical/thermal‐stability of QDs, etc. Aiming to address these issues, many efforts have been done, such as optimizing the structure (e.g., core/shell architecture) of QDs to improve the PLQY for enhanced LSCs' efficiency and tuning optical properties (e.g., band structure engineering) of QDs to reduce re‐absorption effect of LSCs, etc.[[qv: 12c,19]]

In this review, the first part is focused on introducing the operating principle, structure and important technical parameters of LSCs. The second part contains the overview of the synthetic techniques and optical properties of several kinds of eco‐friendly QDs. To be specific, this part focuses on the optimization of re‐absorption, PLQY and long‐term photostability of these eco‐friendly QDs. The latest developments of eco‐friendly QD‐based LSCs are then summarized and the novel design strategies of LSCs are also presented. The existing challenges and prospective outlook of eco‐friendly QD‐based LSCs are proposed in the last part.

## 2 Operating Mechanism of LSCs

The state‐of‐the‐art LSCs are generally defined as a device consisting of an optical waveguide made of transparent polymer incorporated with luminescent materials and coupled with PV cells.[[qv: 10a]] There are various design strategies to fabricate LSCs with many kinds of shapes/sizes, which tends to meet different requirements of implementation on the urban or rural buildings' roofs and windows, indicating that the LSCs hold great promise to popularize the use of solar energy all over the world.^20^

**Figure 1:**
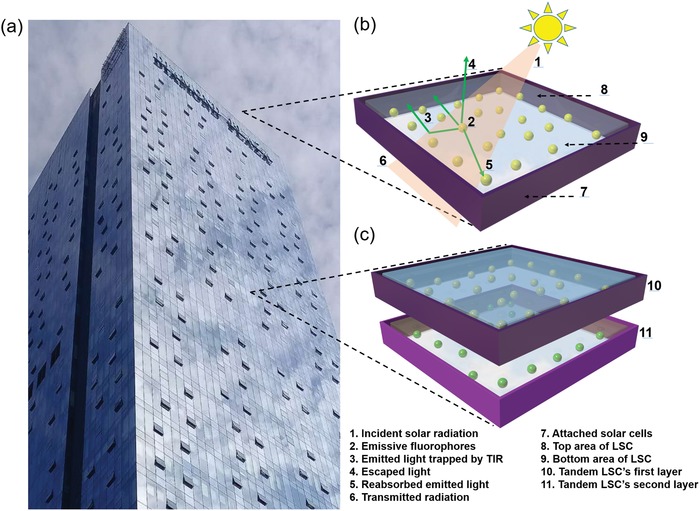
a) Optical image of a scheme of building integrated PV window with LSCs. b) Schematic diagram of a typical LSC and c) a tandem LSC.

**Figure** **1**b illustrates a typical LSC device architecture showing a transparent plastic or glass waveguide with embedded highly emissive fluorophores (e.g., organic dyes, rare‐earth materials, and QDs).[[qv: 17b,21]] The direct/diffused incident light through the large‐area faces is absorbed by the emissive fluorophores, generating the re‐emitted light with longer wavelength, which is guided to the small area face with attached PV cells via TIR for consequent electricity generation.[[qv: 10a,12c,22]]

While the most widely used LSC is single layer architecture, it can be further optimized to enhance the device efficiency by constructing tandem LSCs with multilayered configuration. As depicted in Figure 1c, the tandem LSC is composed of two transparent waveguide layers with incorporated emissive fluorophores. The emissive fluorophores used in first and second layer can absorb different portions of the solar spectrum. As a consequence, the photons with longer wavelength transmitted from first layer can be subsequently utilized by the second layer in the tandem LSCs, thus achieving an overall enhancement of the LSCs' efficiency with respect to single layered counterparts.[[qv: 21b]]

Normally, the optical efficiency and PCE are used to assess the performance of LSCs: The optical efficiency η_opt_ is defined as the ratio of the output power (*P*_out_) from the small area face of LSCs and the input power (*P*_in_) through the top area of the LSC, and is characterized by comparing the photocurrent from the attached PV cells and the photocurrent of identical PV cells under direct solar irradiation.[[qv: 12a,17b,e]]

(1) $$\eta_{\mathrm{opt}}=\frac{P_{\mathrm{out}}}{P_{\mathrm{in}}}\;=\frac{I_{\mathrm{LSC}}}{I_{\mathrm{SC}}\times G}$$

Herein, *I*_LSC_ refers to the measured short circuit current from the PV cell attached to the LSCs. *I*_SC_ represents the short circuit current of identical PV cell under direct solar radiation.

*G* is the geometrical factor of LSCs, which is defined as

(2) $$G\;=\frac{A_{\mathrm{top}}}{A_{\mathrm{edge}}}$$

As shown in Figure 1, *A*_top_ represents the light impending top area of LSC (as shown in Figure 1b) and *A*_edge_ is the smaller edge area coupled with PV cells.

The internal quantum efficiency (IQE) of LSC is simply defined as the ratio of the number of photons emitted from four small faces and total absorbed photons of LSC, as listed below^23^

(3) $$\eta_{\mathrm{int}}=\frac{\eta_{\mathrm{ext}}}{\eta_{\mathrm{abs}}}$$

Here, the external quantum efficiency (η_ext_) is defined as the ratio of number of photons collected from the four small faces and the entire incident photons, the absorption efficiency (η_abs_) is defined as ratio of the number of absorbed photons and total incident photons of LSC.

The PCE of η is another parameter to evaluate the performance of LSCs[[qv: 12a,17b,24]]:

(4) $$\eta \;=\frac{I_{\mathrm{SC}}\times V_{\mathrm{OC}}\times FF}{P\times A_{\mathrm{top}}}$$

In this equation, *V*_OC_ is the open circuit voltage, *FF* refers to the fill factor and *P* is the irradiation intensity. In addition, device stability is also very important to evaluate the performance of LSCs.

With the development of LSCs, the optical efficiency and PCE are still restricted by several loss mechanisms as follows: i) The emitted light from phosphors which is escaped from the waveguide rather than the light guided to the four small faces through TIR. ii) The re‐absorption losses of emitted photons which is caused by small Stokes shift. iii) Low PLQY of emissive fluorophores that limits the efficiencies of LSCs. iv) The absorption range of the majority of emissive fluorophores is limited and unable to absorb all the incident photons and these unabsorbed/transmitted photons subsequently go through the bottom area of LSC (as shown in Figure 1b). v) The decreasing photostability of LSCs under high energy illumination, as a result of fast degradation and decomposition of embedded phosphors.[[qv: 10a,12c,25]] Acting as a key component of LSCs, the stability or durability of waveguide (host materials) should be emphasized as well, which is another significant factor influencing the performance of the LSCs. The waveguide should be also favorable to disperse luminophores, compatible to maintain their optical properties (e.g., PLQY) and show a high tolerance to structural degradation, thus resulting in a highly stable LSC device under long‐term solar illumination.^26^

In order to overcome these loss mechanisms of LSCs, novel emissive fluorophores with outstanding optoelectronic properties including high PLQY, large Stokes shifts, optimal absorption/emission spectrum range and long‐term photostability are highly desired to achieve high efficiency LSCs, which hold great promise in future net‐zero building‐integrated photovoltaic applications.

## 3 Synthesis and Optical Properties of Eco‐Friendly QDs

Semiconducting QDs are nanocrystals with typical diameters ranging from 2 to 20 nm.[[qv: 16c]] Upon the size of QDs reaches nanometer scale, the size limitation could induce the size effect, quantum confinement effect, surface effect, and macroscopic quantum tunneling effect.^27^ Therefore, the properties of these nanocrystals are quite different from those in macroscopic and microscopic systems.^28^ Particularly, the optoelectronic properties of QDs is featured by quantum confinement effect.^29^ Such quantum confinement effect results in the conversion of continuous energy band to discrete energy levels for enlarged band gap of QDs, which further affect their optical absorption and emission spectrum.[[qv: 16c]] For example, the blueshift of QDs' optical absorption spectra occurs when the size of QDs decreases.^30^ As a result, the optical properties of QDs are able to be tuned by controlling their size, shape and composition, leading to tunable absorption/emission spectrum covering UV–vis–NIR region that matches the solar spectrum and is promising for solar energy techniques.

In the last few years, QDs have been demonstrated as excellent emissive fluorophores and were widely used to fabricate high performance LSCs.[[qv: 17e,21a]] However, most of these high‐efficiency LSCs are based on QDs with heavy metals such as CdSe/ZnS QDs, PbS/CdS QDs, Pb‐based perovskite QDs, etc.[[qv: 18c,31]] For instance, Meinardi et al. fabricated a large‐area CdSe/CdS core/shell QD‐based LSCs with no re‐absorption loss, which showed an optical efficiency higher than 10%.^32^ Shcherbatyuk et al. synthesized PbS QDs as emissive fluorophores for LSCs which achieved integrated optical efficiency as high as 12.6%.^33^ All of these results indicate that heavy metal‐based QDs possess excellent optical properties to enhance LSCs' performance, while their intrinsic toxicity is harmful to human's health and natural environment, thus limiting their real‐life usage. The trade‐off between high‐efficiency and environment protection of these heavy metal‐based LSCs is still a major issue for future practical usage and commercialization. Therefore, using eco‐friendly QDs have attracted growing attention for LSCs fabrication. Accordingly, this section focuses on the synthetic methods and optical properties of heavy metal‐free, biocompatible, and environment‐friendly QDs.

### 3.1 Unary Eco‐Friendly QDs

#### 3.1.1 Carbon QDs

Carbon QDs are a promising class of colloidal nanomaterials which have attracted increasing attention recently since they are environmentally friendly, cost‐effective and easy to synthesis.^34^ Carbon QDs are able to achieve tunable band gap by using surface functionalization and passivation, rendering their great potential for various optoelectronic devices such as solar cells, light‐emitting diodes (LEDs) and LSCs.[[qv: 18a,b,34a,c]] Generally, the synthetic methods of carbon QDs include electrochemical carbonization, microwave irradiation and hydrothermal/solvothermal approaches etc. For instance, a facile electrochemical method was developed to prepare carbon QDs with size‐dependent PL properties, while the QY of these carbon QDs is very low.[[qv: 34c]] In this aspect, Liu's group reported a one‐step solvothermal approach to synthesize oil‐soluble carbon QDs with QY as high as 53% (excited at wavelength of 360 nm) that is able to be maintained for 2 months.[[qv: 34b]]

Carbon QDs usually show unique and tunable optical properties (e.g., PL emission). Jiang and co‐workers used a facile solvothermal method to prepare three types of carbon QDs with red, green and blue PL emission under single‐wavelength UV light by using three different carbon sources. These three primary colors make these carbon QDs promising for full‐color display applications. It is assumed that the tunable PL emission are derived from the different amount of N and the size of carbon QDs.^35^

Surface‐states engineering is an effective way to modulate the optoelectronic properties of carbon QDs. Pang's group developed a controllable wet oxidation approach to synthesize carbon QDs with tunable PL emission. By changing the degree of surface oxidation and sizes of carbon QDs, they successfully prepared carbon QDs with various colors of PL emission. It is proposed that the fluorescence of carbon QDs is derived from the emission of surface‐states.^36^

**Figure 2:**
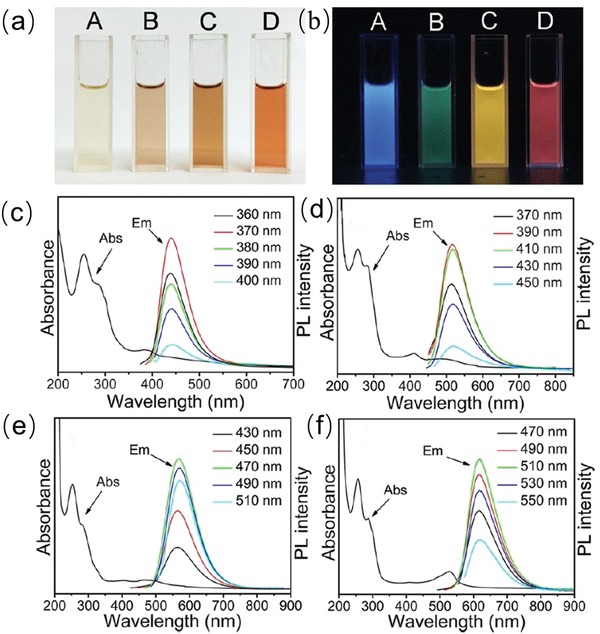
Optical images of four typical samples A (blue), B (green), C (yellow), and D (red) in aqueous solution under a) daylight and b) UV light (right image on the top). c–f) Corresponding absorption and excitation (see the inset denotation)‐dependent PL spectra in the bottom four images (A, B, C, D). Reproduced with permission.^37^ Copyright 2016, American Chemical Society.

Ding et al. synthesized carbon QDs with controllable PL emission and relatively high QY in water via hydrothermal approach with silica column chromatography separation. As‐prepared carbon QDs show tunable, bright and stable PL emission in gradient colors ranging from blue to red under a single UV light excitation. **Figure** **2** shows the optical images of four typical samples A (blue), B (green), C (yellow), and D (red) in aqueous solution under daylight (left image on the top) and UV light (right image on the top). Their absorption and excitation (e.g., see the inset denotation)‐dependent PL spectra are exhibited in the bottom four images. It is demonstrated that carbon QDs in each sample have similar absorption spectra and PL emission centers. The PL emission of each sample are derived from the absorption peak in the long‐wavelength region (i.e., 383, 410, 488, and 528 nm for sample A, B, C, and D, respectively).

These peaks represent different surface‐states of these four types of carbon QDs. Due to that these carbon QDs possessed analogous size distribution and carbon core structures, the displayed redshift of their emission peaks from 440 to 625 nm was attributed to a narrowed band gaps resulted from increasing interaction of oxygen species with surface groups and structures. The surface states are demonstrated to be the critical factor for the tunable optoelectronic properties of carbon QDs.^37^

Shen's group synthesized carbon QDs exhibiting long‐wavelength (orange) PL emission as well as relatively high QY by using the solvothermal method. This work concluded that surface states engineering via surface metal‐cation‐functionalization leads to reduced overlap between absorption and emission spectra of carbon QDs, which is favorable for long‐wavelength orange emission. They speculated that surface metal‐cation‐functionalization could lift the Fermi level of the carbon QDs and result in decreased self‐absorption and improved orange emission. These carbon QDs with small re‐absorption are favorable for solar energy applications such as LSCs.^38^

However, the carbon QDs still encounter some tough challenges, for example, though various methods have been proposed toward the synthesis of carbon QDs, well‐defined structure and controlled sizes are hardly available yet. As for the optical properties, the carbon QDs did not show much advantages over commercial modern dyes in the application of LSCs. For example, the best emitting carbon QDs are actually UV‐blue absorbers and possess all typical feature of dyes. Furthermore, their luminescent mechanism is still not fully understood to date, which needs further comprehensive investigation.[[qv: 34c,39]] Taking these challenges into consideration, there presents much room for the improvements of synthesis and optical properties of carbon QDs. Future research effort can focus on synthesizing carbon QDs in a facile and green method with well‐defined structure and size and excellent optical parameters (e.g., large Stokes shift) for LSCs' applications.

#### 3.1.2 Si QDs

Si QDs exhibit excellent properties including nontoxicity, good photostability, surface tailor‐ability and cost‐effective preparation methods, etc.^40^ These QDs are indirect bandgap semiconductors which differ from conventional heavy metal element‐based QDs. Due to such indirect bandgap structure of Si, the radiative recombination across the bandgap could lead to inefficient emission.^41^ Surface engineering of Si QDs is an effective approach to obtain outstanding optical properties (e.g., size‐tunable luminescence) and long‐term photostability.^42^

Various synthetic methods of Si QDs have been reported in recent years including electrochemical etching of bulk Si, chemical solution based precursor reduction, laser pyrolysis, etc.^43^ For instance, Sato et al. prepared Si QDs with controlled emission wavelength by etching the Si powder with ultrasound and the combination of HNO_3_‐HF.[[qv: 43e]] However, the HF concentration for etching is very high and may cause safety risk and limits the production of Si QDs.[[qv: 43e]] In this respect, Manhat's group have used a one‐step technique employing low‐melting solids as reaction media to synthesize large amount (tens of milligrams per batch) of water‐soluble Si QDs with average size of 4–5 nm.^44^

**Figure 3:**
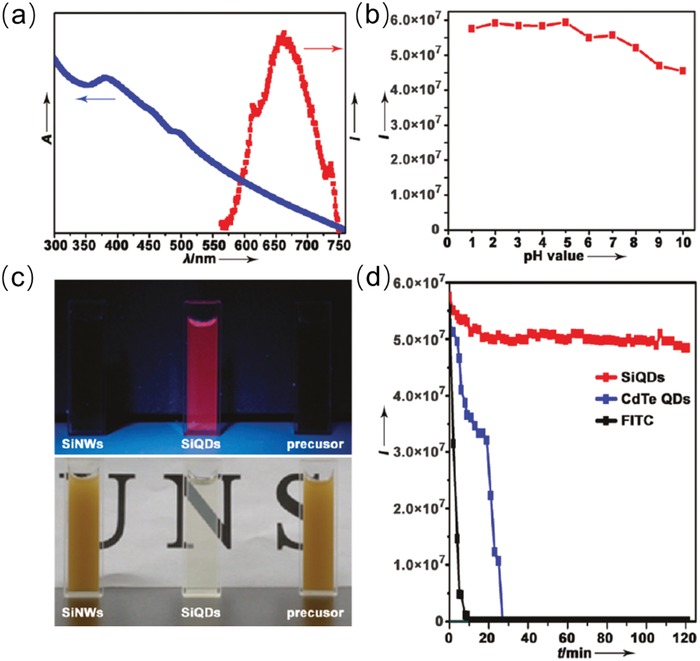
a) Absorption and emission spectrum of as‐synthesized Si QDs. b) PL intensity of Si QDs as a function of varying pH value. c) Photographs of aqueous solutions under 365 nm excitation (up) or ambient illumination (bottom) dispersed with reaction precursors (right), as‐synthesized Si QDs (middle), and SiNWs (left). d) The comparison of photostability among as‐synthesized QDs, CdTe QDs, and FITC. Reproduced with permission.[[qv: 40a]] Copyright 2011, American Chemical Society.

However, Si QDs generally suffer from some deleterious losses, e.g., the luminescence of Si QDs is sensitive and can be quenched with variation of pH values during synthesis.[[qv: 40b,45]] In this regard, He et al. adopted a facile microwave‐assisted one‐pot synthetic method to synthesize water‐dispersible, and pH‐/photostable Si QDs which used Si nanowires (SiNWs) and glutaric acid as precursors.[[qv: 40a]] **Figure** **3**a presents the absorption and PL spectrum of as‐prepared Si QDs, which exhibits an obvious absorption peak and a symmetrical emission peak at 660 nm. Particularly, these Si QDs displays ultra pH‐stability where the PL intensity mildly decrease with changing of pH value, and maintained 85% of its initial value with pH ranging from 1 to 10 (as shown in Figure 3b). This high pH‐stability was caused by glutaric acid which offers dicarboxylic ligands and protective shell for Si QDs. Besides, lots of glutaric acid with hydrophilic carboxylic groups on the Si QDs' surface resulted in outstanding aqueous dispersibility and the aqueous solution of as‐synthesized Si QDs was extremely transparent under ambient light (Figure 3c). The protective ligands shell also leads to superior photostability of Si QDs. According to the stability test, i.e., their PL intensity variation as a function of time in Figure 3d, Si QDs possess the highest photostability with respect to the CdSe QDs and photostable fluorescein isothiocyanate (FITC).[[qv: 40a]]

Wu et al. pointed out that Si QDs' instability under UV illumination is caused by glowing dangling bonds on QD's surface, which leads to nonradiative recombination of Si QDs for reduced PLQY.^46^ In this perspective, they studied the photostability of hydrosilylated, nonthermal plasma synthesized Si QDs under 365 nm excitation. It was found that a 20% reduction in PLQY within 4 h of UV irradiation. Based on these understanding, pursuing excellent surface treatment of Si QDs' surface is a feasible way to achieve outstanding photostability.

Rebecca et al. used nonthermal plasma reactor to produce Si QDs and proved that hydrogen or deuterium atoms passivation can decrease the dangling bonds defects on the surface of Si QDs to obtain high PLQY.^47^ In their experiments, the injection of hydrogen and argon caused the PLQY ≈52 and 12%, respectively, which demonstrated the significance of surface passivation in improving PLQY of Si QDs. Specifically, the PLQY changes along with different injection volume of hydrogen from 0%‐100%. With the increase of hydrogen injection, the hydrogen's coverage on the Si QDs surface was growing so that more dangling bonds states were passivated for enhanced PLQY.^47^ It is concluded that exploring one synthetic technique to achieve the highest hydrogen coverage on Si QDs' can largely enhance the PLQY of Si QDs.

The optical absorption and emission spectra of Si QDs can be tuned from visible to the NIR region by control of the particle size, and their PLQY can be improved to exceed 60%.^48^ While relatively high PLQY of Si QDs has been achieved, it is still challenging to design and synthesize Si QDs with near‐unity QY.^49^ The mechanism of influence of the sizes and type of surface ligand on the optical properties of Si QDs have not been fully discovered.^50^ In addition, the Si QDs suffer from broad size distribution and scattering of emitted light which deteriorate the optical properties.^51^ Owing to these challenges, developing novel facile and controllable synthetic methods for monodispersed Si QDs is desirable. High quality Si QDs with high PLQY and tunable emission spectra, etc., are supposed to be suitable luminescent materials for LSCs' application.

### 3.2 Binary Eco‐Friendly QDs

Unlike widely used toxic Pb and Cd‐based binary QDs such as PbS, PbSe, CdS, CdSe, etc., there are several emerging binary eco‐friendly QDs including Ag_2_S, Ag_2_Se, CuS, CuSe, InP, etc., which are more applicable in future practical QD‐based optoelectronic devices.^52^

Ag_2_S QDs are one of the typical binary eco‐friendly QDs with outstanding optical properties especially in the NIR region. These NIR QDs have been studied for various applications including bioimaging and solar cell.^53^ Many synthetic methods have been developed to synthesize Ag_2_S QDs, examples include thermal decomposition, hot injection and single source precursor method etc.^54^ For instance, Du et al. synthesized monodisperse Ag_2_S QDs by using a single source precursor approach, i.e., decomposition of [(C_2_H_5_)_2_NCS_2_Ag] in octadecylamine, and 1‐octadecene and oleic acid. As‐prepared Ag_2_S QDs exhibited a size of 10.2 ± 0.4 nm with optical NIR emission at ≈1060 nm under 785 nm excitation, indicating the potential applications of these nontoxic QDs for NIR optoelectronic devices.^55^ It is noted that the high mobility of Ag^+^ ions in Ag_2_S crystal structure can give rise to abundant cation vacancies and defect states, which deteriorate the PLQY of QDs and is difficult to be addressed via routine synthetic approaches. Exploring an effective method for suppressed intrinsic defects of Ag_2_S QDs is essential to optimize their optical performance.^56^ Besides, possessing tunable optical properties and “green” feature, binary Cu_2_Se QDs have also been studied in photovoltaics and photonic devices such as optical filters.^57^ These QDs can be easily synthesized by using hot injection and solvothermal methods etc.^58^

InP QDs are another typical binary environmental‐friendly QDs with an exciton Bohr radius of about 10 nm and a bulk band gap of 1.35 eV, enabling the tunable emission wavelength from visible to NIR region by varying its particle size.^59^ Importantly, the PLQY of InP QDs can exceed 70% by coating with specific wide band gap shell materials. Owing to these two attractive optical properties, InP QDs hold great potential in photonic devices.^60^

Previously, strongly coordinating solvents such as trioctylphosphine oxide (TOPO) and mixture of TOPO/trioctylphosphine were used to synthesize InP QDs, while it was time‐consuming (several days) to grow these QDs.^61^ Peng et al. then employed carboxylates in noncoordinating solvent for synthesis of InP QDs, which is fast and can largely improve their optical properties.^62^ This synthetic method can separate nucleation from growth stages, which enables production of monodisperse InP QDs. Woo Seuk et al. produced high‐quality InP QDs by a facile method which injected a unusual phosphorus source of P(N(CH_3_)_2_)_3_ at high temperature other than P(TMS)_3_.^63^ P(N(CH_3_)_2_)_3_ can be activated by oleylamine and then accelerate the formation of InP QDs.

**Figure 4:**
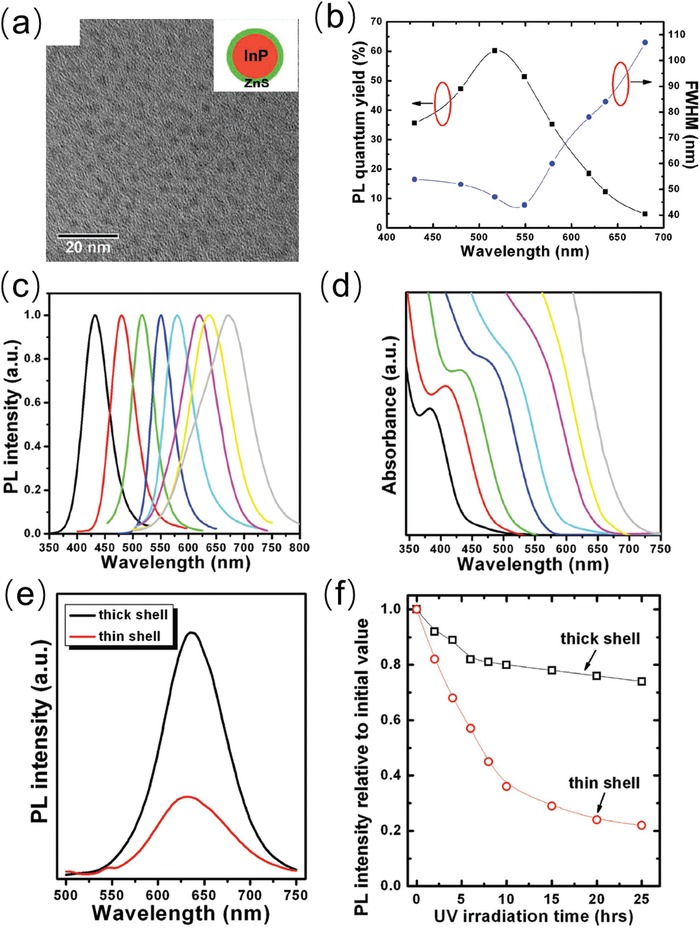
a) TEM image of as‐synthesized InP/ZnS with low magnification. b) PLQY and FWHM evolution of InP/ZnS core/shell QDs, c) PL spectra and d) absorption spectra with varying ratios of InP:ZnS. e) PL intensity and f) photostability test of as‐synthesized InP/ZnS QDs with thin and thick ZnS shell. Reproduced with permission.[[qv: 64d]] Copyright 2012, John Wiley and Sons.

Formation of core/shell structure is a suitable surface modification method to improve optical properties (e.g., PLQY) and photostability of InP QDs.^64^ Sun et al. used a simple solvothermal approach to synthesize InP/ZnS QDs with very high PLQY over 60%.[[qv: 64d]] As‐synthesized InP/ZnS QDs exhibited narrow size distribution, as shown in the TEM image (**Figure** **4**a). The absorption and emission spectra of as‐synthesized InP/ZnS QDs can be tuned by varying the molar ratio of InP:ZnS, which is beneficial to achieve optimized Stokes shift. As exhibited in Figure 4c,d, the absorption and emission spectra redshifted and were tuned from UV to NIR region with increasing InP:ZnS molar ratio (from 1:2 to 16:1).[[qv: 64d]]

With molar ratio of InP:ZnS reaching 1.2:1, InP/ZnS QDs obtained the optimal PLQY of 60.2% (shown in Figure 4b). While the PLQY decreased with further increasing of InP/ZnS molar ratio, which is ascribed to that the thin ZnS shell could not offer an effective surface passivation of InP QDs. To address this issue, an additional shelling of ZnS is coated on primary InP/ZnS QDs to further improve the PLQY from 12.3% to 38.1% (Figure 4e). As for the photostability, a rapid decrease of PL intensity occurred in the case of thin shell InP/ZnS QDs, however, the thick shell InP/ZnS QDs showed slight decrease for even 25h UV irradiation due to effective ZnS shell passivation (Figure 4f). All of these synthetic methods and surface engineering approaches can optimize the optical properties of InP, which proves its feasibility in acting as highly emissive materials in optoelectronic devices including LEDs and LSCs. Nevertheless, more stable phosphorus precursors should be developed for InP QDs synthesis to avoid broad size distribution and emission line width.^65^ The interfacial layer engineering of InP‐based core/shell QDs should be studied to better passivate the defects for superior PLQY.[[qv: 65b]] It is also necessary to balance the stability and optical properties of InP core/shell‐based QDs for optoelectronic applications.

### 3.3 Ternary Eco‐Friendly QDs

Conventional ternary QDs such as CdTe*_x_*Se_1−_ *_x_*, CdSeS, Zn_1−_ *_x_*Cd*_x_*Se, etc., are usually made of heavy metal elements which is not eco‐friendly. So far, the ternary I–III–VI_2_ chalcopyrite compound QDs such as CuInS_2_, CuInSe_2_, CuInSe*_x_*S_2−_ *_x_*, and AgInS_2_, which are free of heavy metals and eco‐friendly, have received increasing attention in the last few decades.^66^ These environment‐friendly ternary chalcopyrite QDs present superior optical properties in solar energy techniques including: i) The absorption spectra of these types of QDs cover UV–vis and NIR region, which match solar spectrum well and are suitable for light harvesting; ii) Their optical absorption coefficients is relatively high; iii) The PLQY of these QDs can reach to almost 80% in the visible to NIR region by inorganic shell passivation, such as ZnS shelling.^67^

CuInS_2_ QDs are typical ternary eco‐friendly QDs with mature synthetic approaches including hot injection techniques, thermal decomposition, solvothermal methods, etc.^68^ For example, Liu et al. presented a simple and cost‐effective hydrothermal synthesis method to synthesize CuInS_2_ QDs by using mercaptopropionic acid (MPA) as the stabilizer.[[qv: 66a]] Using this synthetic method, they synthesized stable CuInS_2_ QDs with tunable absorption and emission.[[qv: 66a,68b]] Li and his co‐workers developed a facile solvothermal approach to synthesize monodisperse CuInS_2_ QDs with sizes smaller than 5 nm as well as very narrow size distribution (7–11%).[[qv: 66c]]

**Figure 5:**
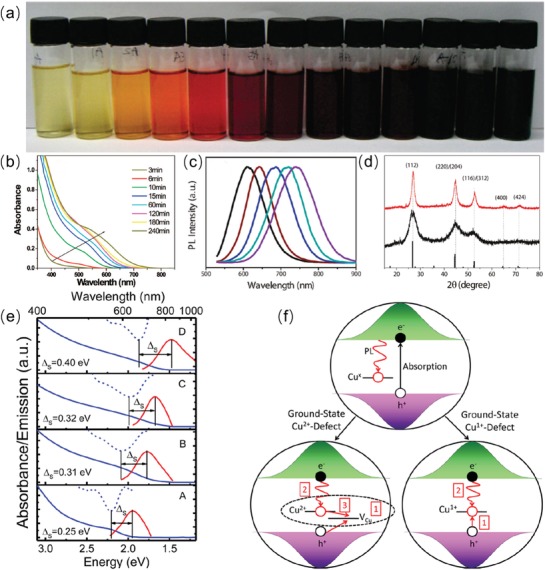
a) Photographs of CuInS_2_ QDs heated at 240 for 1, 3, 6, 10, 15, 20, 30, 60, 120, 180, 240, 300, and 360 min, respectively (from left to right). b) Absorption spectra of CuInS_2_ QDs taken from the reaction solution heated at 240 °C for 3, 6, 10, 15, 60, 120, 180, and 240 min, respectively. Reproduced with permission.[[qv: 68b]] Copyright 2008, American Chemical Society. c) PL spectra of CuInSe_2_/ZnS QDs with different core sizes. d) XRD of CuInSe_2_ QDs synthesized at 180 °C (black line) and 200 °C (red line). Reproduced with permission.^71^ Copyright 2011, American Chemical Society. e) Absorption and emission spectra of as‐synthesized CuInS_2_ QDs with four different sizes (increasing from bottom to top). f) Intra gap emission of CuInS_2_ QDs induced by Cu‐defect‐related mechanisms. Reproduced with permission.^72^ Copyright 2017, American Chemical Society.

The optical properties of colloidal CuInS_2_ QDs are dependent on their size, shape, structure, and surface states, etc.[[qv: 67e,68b,69]] Zhong et al. established a simple synthetic method of CuInS_2_ QDs using the copper (I) acetate, indium (III) acetate, and dodecanethiol as precursors to prepare CuInS_2_ QDs. The size and shape of as‐synthesized CuInS_2_ QDs can be easily controlled by controlling the reaction temperature and time. The sizes of these CuInS_2_ QDs are around 2–5 nm with various experiment conditions. Their absorption and emission spectrum are also tunable ranging from 550–760 nm and 600–750 nm, respectively. The reaction time determines the growth dynamic of CuInS_2_ QDs. As shown in **Figure** **5**a, with increasing reaction time, the color of CuInS_2_ QDs changes from bright yellow to dark brown. Meanwhile, the absorption spectra of CuInS_2_ QDs continuously broaden with increased reaction time (Figure 5b). Furthermore, it is found that surface‐related recombination, direct exciton recombination, and donor‐acceptor recombination are the three main recombination pathways of CuInS_2_ QDs' PL emission, and the donor‐acceptor recombination is the dominant process.[[qv: 68b]]

CuInSe_2_ QDs are another typical ternary eco‐friendly QDs with a bulk band gap about 1.04 eV and comparatively large exciton Bohr radius about 10.6 nm, which have demonstrated efficient luminescent emitters in the NIR region.^70^ Zhong et al. used an organometallic method to prepare CuInSe_2_ QDs with tunable PL emission.^71^ As compared to CuInSe_2_ core QDs, inorganic ZnS shell was then employed to synthesize CuInSe_2_/ZnS core/shell QDs with enhanced PLQY up to 26%. More importantly, the CuInSe_2_/ZnS QDs' PL emission can be tuned ranging from 600–850 nm by varying the size of CuInSe_2_ QDs, as shown in Figure 5c. X‐ray diffraction (XRD) patterns in Figure 5d demonstrates that these CuInSe_2_ QDs possess stable chalcopyrite phase structures at room temperature.^71^

Klimov et al. studied the size dependent Stokes shift of CuInS_2_ QDs.^72^ Figure 5e presents the absorption and emission spectra of as‐synthesized CuInS_2_ QDs with four different sizes. The Stokes shift (Δ*s*) enlarged from 0.25 to 0.40 eV with the increasing size of CuInS_2_ QDs. This apparent large Stokes shift of CuInS_2_ QDs may originated from the involvement of intra gap states during emission stage (Figure 5f, top). Moreover, the intra gap state was related to the Cu*^x^* defect including Cu^2+^ and Cu^1+^ (Figure 5f bottom left and right).^72^

For the PLQY, lots of efforts have been made for CuInS_2_‐based QDs. Kim et al. synthesized CuInS_2_/ZnS QDs with a high PLQY of 65% through a cation exchange method.^73^ Yang et al. synthesized CuInS_2_/ZnS QDs with an efficient passivation via overcoated ZnS layer, achieving a remarkable PLQY in the range of 68% to 78%.^74^

AgInS_2_ QDs are also one of representative ternary “green” QDs with direct band gap and high optical absorption coefficients from visible to NIR region.^75^ , rendering their widespread usage in various photovoltaic devices.^76^ Numerous synthetic methods have been developed to synthesize AgInS_2_ QDs such as thermal decomposition, etc.^77^ Chang et al. took a facile two‐step method to synthesize AgInS_2_ QDs and AgInS_2_/ZnS QDs by injecting ZnS precursors into AgInS_2_ QDs, which resulted in AgInS_2_ QDs and AgInS_2_/ZnS core/shell QDs with high PLQY of 22% and 60%, respectively.[[qv: 75a]]

These results suggest that ternary eco‐friendly QDs show excellent optical properties including high PLQY and large Stokes shift that are promising candidates for LSCs' application. However, these ternary chalcopyrite QDs still needs to overcome several drawbacks, such as relatively poor photostability.^78^ Ternary I−III−VI_2_ QD intrinsically possess abundant defects which allow the tunable PL spectra and feasibility of alloying, but it also promotes inhomogeneous composition of QDs that results in a variation of optical band gap and broadening of the PL peak.^79^ It is hence desirable to the comprehensively investigate their luminescent properties. Although these ternary QDs are regarded as a low‐toxicity replacement for heavy metal‐based QDs, the in‐depth understanding of their toxicity should be developed to be fully realized in the application of optoelectronic devices including LSCs.[[qv: 78b]]

Despite the outstanding optical properties of unary, binary, and ternary eco‐friendly QDs, the overall durability and stability of these QDs is another significant factor to be addressed. In general, colloidal QDs were very sensitive to surrounding environment such as light, oxygen, moisture, and temperature.[[qv: 42d,80]] The photo/thermal/chemical‐stability of QDs can be improved by selecting appropriate surface ligand and using shell coating and oxide overcoating strategies.^81^

The ligands on QD's surface can stabilize the QD surface to avoid weak dangling bonds. Using strong binding between the QDs and anchor groups is an effective approach to improve the photo/thermal‐stability of the QDs. For instance, QDs capped with alkylthiol ligands show better thermal‐stability than QDs with carboxylic acid ligands.^82^ The growth of core/shell structure is another approach to efficiently suppress the surface defects/traps of QDs and largely enhance the stability of QDs, examples include the eco‐friendly InP/ZnS, AgInS_2_/ZnS core/shell QDs as mentioned above.[[qv: 64d,75a]] The stability of QDs can be also improved by interfacial shell engineering and increasing shell thickness. The use of interfacial shells enhances the stability of QDs by alleviating the lattice mismatch between the core and shell. For example, eco‐friendly InP//GaP/ZnS exhibit long‐term photostability with slight PL intensity degradation over 100 h under UV light illumination.^83^ Heavy metal‐free core/thick shell QDs, such as “giant” CuInSe_2_/CuInS_2_ QDs showed pronounced stability under corrosive condition.^30^ Moreover, oxide overcoating has demonstrated effective technique to prevent the QDs from oxygen and moisture.[[qv: 81d,84]] By using In_2_O_3_ overcoating, the PLQY of InP/ZnS QDs can be boosted from 48% to 59% and such In_2_O_3_‐coated InP/ZnS QDs showed largely improved photostability as compared to bare InP/ZnS after long‐term UV illumination.

## 4 Eco‐Friendly QD‐Based LSCs

### 4.1 Unary Eco‐Friendly QD‐Based LSCs

#### 4.1.1 Carbon QD‐Based LSCs

Carbon QDs, which are comprised of earth‐abundant and nontoxic elements (C, N, and O), have exhibited unique merits including facile synthesis, cost‐effectiveness and high stability.[[qv: 34b,85]] Carbon QDs also showed tunable optical properties and high PLQY, rendering their great potential in LSCs, which may significantly enhance the device performance including optical efficiency, PCE, photostability, etc.^86^

**Figure 6:**
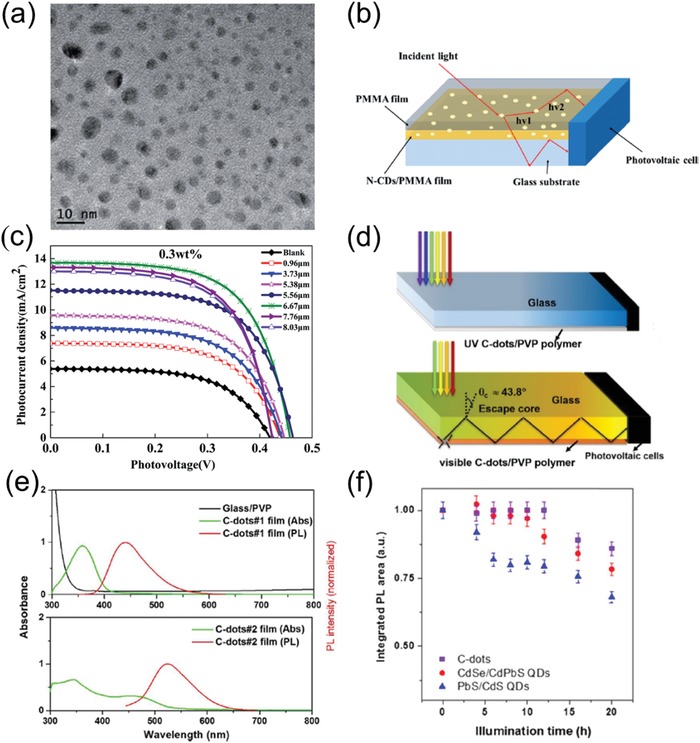
a) Typical TEM image of N‐doped carbon QDs. b) Schematic diagram of the LSCs consisting of a polymer matrix with N‐doped carbon QDs. c) The *J*–*V* curves of N‐doped carbon QDs/PMMA thin film LSCs with varying film thicknesses. Reproduced with permission.[[qv: 18a]] Copyright 2017, Royal Society of Chemistry. d) Schematic illustration of tandem thin‐film LSCs based on UV carbon QDs and visible carbon QDs. e) The absorption and PL spectra of UV carbon QDs (top) and visible NaOH treated carbon QDs (bottom)‐based LSCs as well as the absorption spectrum of PVP/glass substrate. f) The variation of integrated PL area of carbon QDs, CdSe/CdPbS QDs, and PbS/CdS QD‐based LSCs under UV illumination. Reproduced with permission.[[qv: 18b]] Copyright 2017, Elsevier.

Li et al. developed an LSC device utilizing eco‐friendly N‐doped carbon QDs as the phosphors because N‐doping of carbon QDs have already been proved to be a suitable method for improved optical properties such as high PLQY.[[qv: 18a,87]] Poly(methyl methacrylate) (PMMA) polymer were selected as the host material of waveguide, because PMMA possesses several suitable properties for LSCs' fabrication, such as high refractive index ≈1.49, high transparency (92%) in visible light region, good stability and durability under various ambient conditions.[[qv: 26a]] In the fabrication processes of LSC, N‐doped carbon QDs were used as optical emitters, which were mixed with PMMA polymer and spin‐coated on glass substrates. **Figure** **6**a displayed the TEM images of as‐synthesized N‐doped carbon QDs, showing spherical shapes without significant aggregation. Figure 6b shows the schematic diagram of the N‐doped carbon QD‐based LSCs. It is found that the absorbance of N‐doped carbon QDs/PMMA enhanced with increasing film thickness, i.e., improved amount of N‐doped carbon QDs. Figure 6c exhibits the *J*–*V* characteristic curves of N‐doped carbon QDs/PMMA thin film LSCs with various film thicknesses. The N‐doped carbon QDs/PMMA LSCs obtained the optimized performance with film thickness of 6.67 µm, showing highest optical efficiency of 4.75% and overall PCE of 3.94%. Furthermore, this type of N‐doped carbon QD‐based LSCs exhibit long‐term photostability.[[qv: 18a]] The successful fabrication of such carbon QD‐based LSC provides the guidelines for LSC's industrial purposes.

Zhou et al. reported other kinds of environmental‐friendly colloidal carbon QD‐based LSCs.[[qv: 18b]] Different types of carbon QD‐based LSCs were fabricated by spin‐coating the mixture of water‐soluble carbon QDs and polyvinylpyrrolidone (PVP) on the glass substrate or combining oil‐soluble oleylamine (OLA)‐treated carbon QDs into photopolymerized poly(lauryl methacrylate) (PLMA).[[qv: 26a]] Figure 6f presents the stability measurements (i.e., integrated PL area as a function of UV illumination time) of carbon QDs, CdSe/CdPbS QDs and PbS/CdS QD‐based LSCs. All of these QD‐based LSCs exhibited outstanding device stability after long‐time UV illumination (over 12 h), while the CdSe/CdPbS and PbS/CdS QD‐based LSCs showed significant loss with further UV illumination as compared to carbon QDs LSCs. The carbon QDs LSCs also showed negligible degradation after storage in air for more than 4 months. All of these results demonstrate the ultra‐high stability of carbon QDs LSCs.[[qv: 18b]]

A tandem carbon QD‐based LSC was further developed in this work, as shown in Figure 6d. The first layer of the tandem thin film LSCs were fabricated using UV carbon QDs and the second layer was fabricated employing NaOH treated visible carbon QDs and untreated C‐dots. As depicted in Figure 6e, the absorption spectra of UV carbon QDs range from 300 to 400 nm and exhibit large Stokes shift with reduced loss of re‐absorption and a remarkable PLQY of 60%. As compared to the UV carbon QDs, the second layer of visible carbon QDs possess larger absorption spectra ranging from 300 to 550 nm and also considerable PLQY (≈40%). As a result, the second layer containing visible carbon QDs is able to absorb the escaped emitted visible light from the first layer with UV carbon QDs. Based on this complementary multilayer design, such tandem carbon QD‐based LSC showed an excellent optical efficiency of 1.1%.[[qv: 18b]] The design of the tandem thin film LSCs is innovative which move people's attention of single layer LSCs to tandem LSCs and present a method to develop combinations of different luminescent material‐based LSCs.

**Figure 7:**
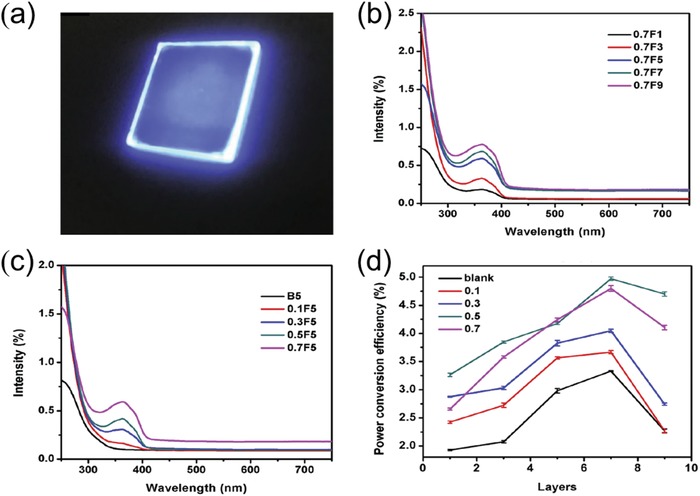
a) Photograph of N‐doped carbon QD‐based LSC under UV illumination. b) Absorption spectrum of N‐doped carbon QD‐based LSCs with different layer numbers ranging from 1, 3, 5, 7, and 9 at the same loading concentration of 0.7 wt%. c) Absorption spectrum of N‐doped carbon QD‐based LSCs with different loading concentration ranging from 0.1, 3, 0.5, and 0.7 wt% at the same layer number 5. d) Graph of optical efficiency of LSCs and loading concentrations of the N‐doped carbon QDs at varying layer numbers of N‐doped carbon QDs/PVP thin film. Reproduced with permission.^88^ Copyright 2018, Elsevier.

Due to the good compatibility between N‐doped carbon QDs and PVP, Wang and his co‐workers fabricated an N‐doped carbon QDs/PVP‐based LSC. The optical performance of the LSCs with diverse layers and concentration of N‐doped carbon QDs was comprehensively studied.^88^ The photograph of this N‐doped carbon QDs/PVP‐based LSC under UV illumination is displayed in **Figure** **7**a, showing concentrated light emitted from the side edges of LSC. Figure 7b shows the broad absorption peak of such N‐doped carbon QD‐based LSCs located at 350–400 nm and the absorbance improved with layer numbers, which is attributed to the growing number of N‐doped QDs in the thin films.

Figure 7c indicates that the absorption intensity also gradually increased with the carbon QDs concentration based on identical layer number of 5. It is vividly shown in Figure 7d that the PCE of LSCs increased with layer number and N‐doped carbon QDs concentration, reaching the maximum value of 4.97% at layer 7 and concentration of 0.5%.^88^ This work also indicates that carbon QDs are promising for fabrication of high performance LSC.

Another investigation by Maria et al. fabricated LSCs based on in situ cross‐linking organosilane‐functionalized carbon QDs (Si‐CND) with high loading contents (25 wt%). Such high loading LSCs can still show a highly stable PLQY up to 45% together with high internal quantum efficiency of ≈22%. Nevertheless, the large re‐absorption losses caused by high loading concentration needs to be further optimized to achieve large‐are LSC devices.^89^

In summary, carbon QD‐based LSCs have emerged as promising candidates for forthcoming net zero energy consumption buildings owing to their eco‐friendly feature, excellent optical properties and long‐term photostability. More investigation of these devices could definitely open the new chapter of LSCs' research and bring bright future for the solar energy industry.

#### 4.1.2 Si QD‐Based LSCs

Si is earth‐abundant, eco‐friendly, and cost‐effective.[[qv: 1a,90]] Si QDs are one of the typical indirect‐band gap semiconductors with very small absorption coefficient at the absorption edge, leading to suppressed re‐absorption losses that is suitable for the state‐of‐the‐art semi‐transparent LSCs.^48^, ^91^

**Figure 8:**
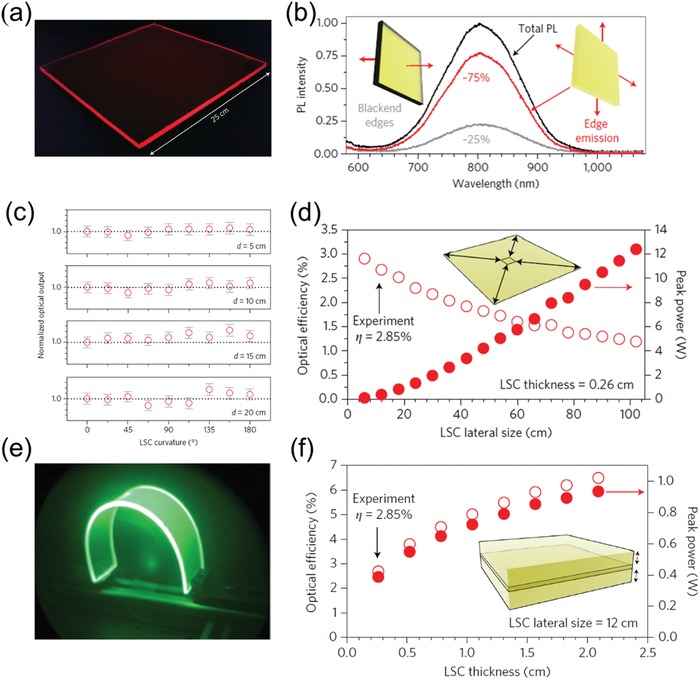
a) Photograph of Si QD‐based LSC under UV illumination. b) PL spectra of Si QD‐based LSCs: black line for total photoluminescent, red line for the edge clear part and gray line for the blocked edge by black paint. c) The optical output as a function of θ with different *d*. d) Monte Carlo ray‐tracing simulations of optical efficiency as a function of LSCs area. e) Photographs of a flexible Si QD‐based LSCs under ultraviolet illumination taken with the ultraviolet‐filtered infrared camera. f) Monte Carlo ray‐tracing simulations of optical efficiency as a function of LSCs thickness. Reproduced with permission.[[qv: 18g]] Copyright 2017, Springer Nature.

Si QD‐based LSCs were proposed by Meinardi et al. in 2017.[[qv: 18g]] Herein, Si QDs emitting at 830 nm were prepared in a nonthermal plasma reactor and subsequently employed as optical emitters in LSCs. **Figure** **8**a presents the picture of as‐fabricated large‐area Si QD‐based LSC under UV illumination, showing concentrated red light emitted from four small faces. Figure 8b illustrated that the luminescence guided to the LSCs' edges is 75%, which matches well with the maximum theoretical light‐trapping efficiency (Snell's law) for suppressed re‐absorption.^92^ A flexible Si QD‐based LSCs was also fabricated (Figure 8e) with bending angle of θ =180∘  as an arch‐like structure. Unexpectedly, as exhibited in Figure 8c, the optical output keeps constant in all angles (from $0^{{}^{\circ}}$ to 180∘) with *d* varies in 5, 10, 15, and 20 cm, which is consistent with the Monte Carlo ray‐tracing simulations, demonstrating that such Si QD‐based LSCs are appropriate to realize architectures with complex curvature. The theoretical results of Si QD‐based LSCs with thickness of 0.26 cm and area of 12 × 12 cm^2^ are shown in Figure 8d, which shows that the optical efficiency decreased with larger LSC lateral area due to synergetic effect of low reabsorption and scattering losses. As illustrated in Figure 8f, the optical efficiency gradually improved with the increasing LSC thickness. According to this theoretical simulation, the highest optical efficiency was up to 7% with the thickness of 2.0 cm.[[qv: 18g]] This investigation paves the way for rational design of eco‐friendly, high performance and flexible LSCs for building integration.

### 4.2 Binary Eco‐Friendly QD‐Based LSCs

**Figure 9:**
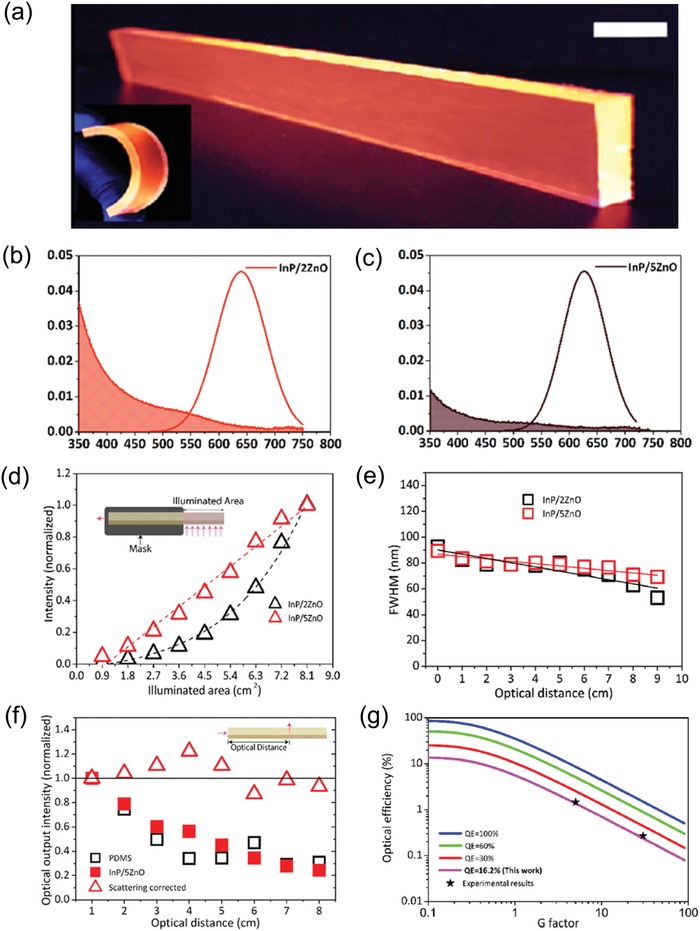
a) Digital photograph of InP/ZnO QD‐based LSC under ambient light (1 cm for the scale bar). Absorption and emission spectra of b) InP/2ZnO QDs and c) InP/5ZnO QDs. d) Optical output as a function of illuminated area and e) FWHM as a function of optical distance of InP/2ZnO and InP/5ZnO QDs‐LSCs. f) The optical output intensity comparison of PDMS and InP/5ZnO QD‐based LSCs, respectively. g) Simulations of optical efficiency from different QE levels. Reproduced with permission.[[qv: 12d]] Copyright 2018, American Chemical Society.

Binary nontoxic InP/ZnO core/shell QDs have recently been used by Sadra et al. to fabricate LSCs, in which the type‐II band alignment of InP/ZnO QDs is utilized to optimize the Stokes shift.[[qv: 12d]] Investigation of InP/ZnO core/shell QDs with various shell thickness have been carried out in this work. The emission peaks of QDs redshifted with increasing shell thickness of ZnO due to that the holes were confined strongly in the core, while the electron tended to delocalize to ZnO shell. As a result, with the increasing of shell thickness, the overlap of absorption and PL spectra became smaller for less re‐absorption (**Figure** **9**b,c).

Figure 9a displays the digital photograph of InP/ZnO QD‐based LSC under UV illumination, showing structural flexibility of as‐fabricated device. Correspondingly, the optical output intensity of InP/2ZnO QDs and InP/5ZnO QD‐based LSCs as a function of illuminated area is shown in Figure 9d. The InP/2ZnO QD‐based LSC showed a near‐exponential growth of optical output with the increasing illuminated area, which was caused by the reabsorption loss in InP/2ZnO‐based LSC. In contrast, the InP/5ZnO QD‐based LSC exhibited a linear response of the output optical power with the increasing illuminated area, which was originated in the negligible reabsorption loss in InP/5ZnO QD‐based LSC. Furthermore, the full width at half‐maximum (FWHM) of as‐fabricated LSCs as a function of optical distance is shown in Figure 9e. With increasing optical distance from 0 to 9 cm, the FWHM of the InP/2ZnO QDs‐LSC decreased at a slope of (3.29 nm/cm), while InP/5ZnO QD‐LSC decreased at a lower slope of 1.80 nm/cm due to smaller reabsorption.

The variation of loading concentration on LSCs' optical efficiency was studied as well. It indicates that the optical efficiency of LSC was improved from 0.28 to 3.22% with increasing QD's loading concentration as a result of enhanced absorption. However, the LSCs become not transparent in high QD's loading concentration, suggesting that it is very significant to balance the light absorption and transmission of these LSCs for their practical applications of solar windows in building integration.

Figure 9g presents that the simulated optical efficiency of LSCs is improved with higher QE of QDs and reduced with increasing G factor. The optical efficiency of this type of LSC reached 1.4% at G factor of 5 and decreased to 0.225% at G factor of 30. As illustrated in Figure 9f, the scattering intensity almost maintained with increasing optical distance, which demonstrated that the scattering loss is the major optical decay of this LSC. It is noted that the thick ZnO shell can efficiently suppress the host material effect of LSC and achieve good photostability of InP/ZnO QDs.[[qv: 12d]]

### 4.3 Ternary Eco‐Friendly QD‐Based LSCs

Ternary eco‐friendly chalcopyrite QDs are emerging alternatives for conventional heavy metal‐based toxic QDs due to their high extinction coefficient, broad light absorption, environment‐friendless, as well as cost‐effective synthesis, etc., which are favorable for fabrication of QD‐based optoelectronic devices.^92^

Theoretically, Hu et al. used Monte‐Carlo ray‐trace simulation to study the optical performance of ternary eco‐friendly CuInS_2_ and CuInSe_2_ QD‐based LSCs.^94^ The effect of LSCs' size and QDs' QY on device performance was studied, it was found that the performance of CuInS_2_ and CuInSe_2_ QD‐based LSCs can be boosted with higher PLQY of QDs due to the improved photon collection efficiency. This theoretical Monte‐Carlo ray‐trace simulation suggests the great potential of ternary green CuInS_2_ and CuInSe_2_ QDs for LSCs fabrication.

**Figure 10:**
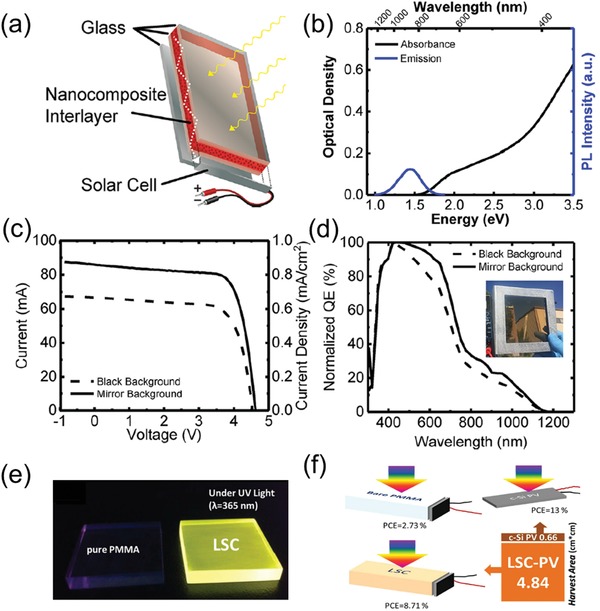
a) Schematic diagram of the laminated glass LSCs. b) Absorption and PL spectra of CuInS_2_/ZnS QDs in solution. c) Certified *I*–*V* curves and d) normalized QE of the LSCs with black background (dashed curve) and mirror substrate (solid curve) under AM1.5 illumination. Reproduced with permission.[[qv: 20a]] Copyright 2018, American Chemical Society. e) Photographs of pure PMMA‐based LSC and CuInS_2_/ZnS‐based LSC illuminated by same UV lamp. f) PCE comparison among different LSC‐PV devices. Reproduced with permission.^95^ Copyright 2015, Springer Nature.

Matthew et al. used a low‐cost heat‐up method to synthesize ternary eco‐friendly CuInS_2_ QDs with ZnS passivation shell and fabricated corresponding QD‐based LSC.[[qv: 20a]] A new structure for LSC is designed and shown in **Figure** **10**a in which a polymer interlayer embedded with CuInS_2_/ZnS QDs is sandwiched between two sheets of low‐iron glass as compared to conventional float glass, leading to threefold improvement of optical efficiency. Figure 10b shows the absorption and emission spectra of as‐prepared CuInS_2_/ZnS QDs, exhibiting large Stokes shift with band‐edge (identified by broad absorption shoulder) at ≈2.0 eV and the emission peak at ≈1.44 eV.

Figure 10c,d displays the *I*–*V* curve and the normalized QE of as‐fabricated LSC with black background (dashed curve) and mirror substrate (solid curve), respectively. It indicates that the as‐fabricated LSC with a mirror substrate exhibit better performance than LSC with a black background. The optical efficiency and PCE of as‐fabricated CuInS_2_/ZnS QD‐based LSCs reached up to 8.1% and 2.9%, respectively. Due to its high capability of integration into facades of building and high efficiency, this kind of laminated glass LSCs could be a new tendency in the future.

Chen et al. have fabricated a ternary eco‐friendly CuInS_2_/ZnS QD‐based LSCs with optical efficiency and PCE reaching up to 26.5% and 8.71%.^95^ A facile one‐step method was employed to synthesize CuInS_2_/ZnS QDs with a PLQY of 81% and large Stokes shift (over 150 nm). The photographs of these two LSCs under the UV light wavelength of 365 nm are shown in Figure 10e and the performance of CuInS_2_/ZnS QD‐based and PMMA‐only‐based LSCs are compared in Figure 10f. The PCE increases from 2.73% for the PMMA‐only LSC to 8.71% for the CuInS_2_/ZnS QD‐based LSC, demonstrating the concentrated function of CuInS_2_/ZnS QDs.^95^

Zn and Al co‐doped ternary eco‐friendly CuInS_2_ QDs were also synthesized to fabricate LSCs by Zhu et al.^96^ As‐synthesized Zn and Al co‐doped CuInS_2_ QDs depicted an obvious peak around 740 nm in NIR region and the PL intensity improved with increasing doping time (10 to 30 min). The enhanced optical properties of Zn and Al co‐doped CuInS_2_ QDs were mainly due to the involving Zn_Cu_ and Al_Cu_ defects that reduces the number of dangling bonds acting as the nonradiative recombination centers. The optical gap of co‐doped CuInS_2_ QDs gradually decreased to 2.60 eV with increasing doping time, this value is far away from the PL emission peak, indicating the large Stokes shift of these doped CuInS_2_ QDs. As a result, the optical efficiency of Zn and Al co‐doped CuInS_2_ QD‐based LSCs reached up to 6.97%.^96^

The QD‐based LSCs are generally comprised of pure QDs without other impurities, while Liu et al. fabricated a CuInS_2_/ZnS QD‐based LSC with SiO_2_ particles acting as the scattering center. Since the light absorption range of c‐Si PV cells is between 400 nm to 900 nm, the role of SiO_2_ particles can be listed as follows: i) Enhancing the PL emission of QDs via scattering the incident light (wavelength below 400 nm). ii) Guiding more incident light and re‐emitted light of QDs to PV cells. Because of the scattering effect induced by SiO_2_ particles, a PCE of 4.20% was achieved in such CuInS_2_/ZnS QD‐based LSC, showing 60.3% improvement with respect to LSC without SiO_2_ particles.[[qv: 18f]]

**Figure 11:**
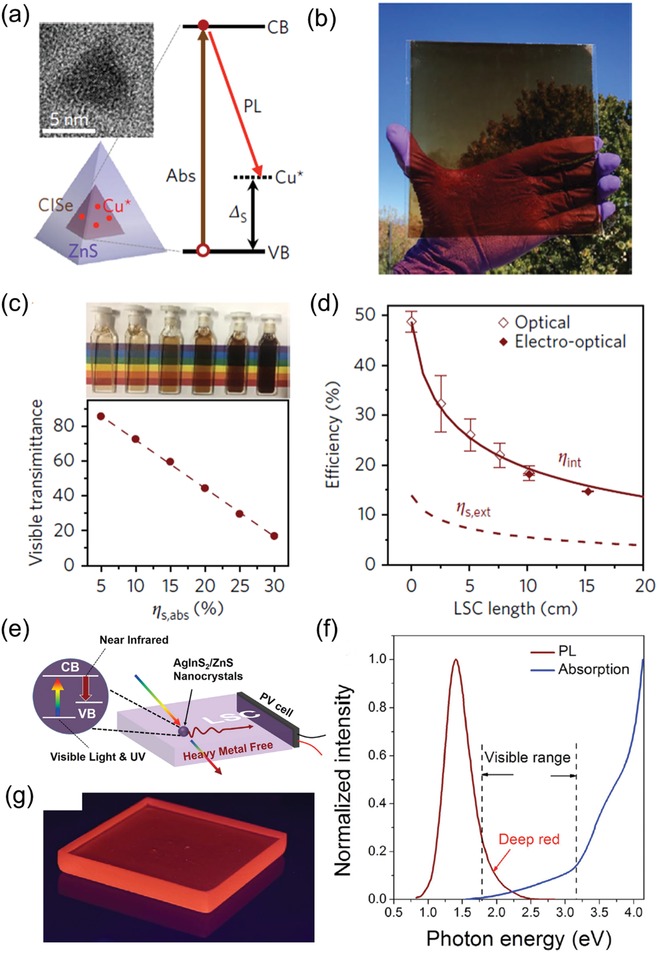
a) Typical TEM image of CuInSe_2_/ZnS QD (top left) and illustration of a single CuInSe_2_ QD (bottom left), and scheme of optical transitions and electronic state (right). b) Optical image of CuInSe_2_ LSC under ambient condition. c) CuInSe_2_/ZnS QDs (in solution) with growing sunlight absorptances (η_s,abs_) and relevant VT. d) η_int_ characterized by the PV (solid diamonds) and integrating‐sphere (hollow diamonds) methods as a function of LSC's length. The brown solid line exhibit the theoretical results. The EQE (η_s,ext_, brown dashed line) is gained by multiplying η_int_ by η_s,abs_ (= 28%) under sunlight illumination. Reproduced with permission.^97^ Copyright 2018, Springer Nature. e) Schematic diagram of the AgInS_2_/ZnS QD‐based LSC. f) Normalized absorption and emission spectra of AgInS_2_/ZnS QD‐based LSC. g) Photograph of AgInS2/ZnS QD‐based LSC under UV illumination. Reproduced with permission.^76^ Copyright 2017, John Wiley and Sons.

Klimov et al. fabricated an LSC based on ternary eco‐friendly CuInSe_2_/ZnS QDs.^97^ The PL emission of CuInSe_2_/ZnS QDs is derived from the transition between a conduction‐band electron and a localized hole located at Cu‐related defect (**Figure** **11**a, right).^98^ Consequently, the Stokes shift of the as‐synthesized CuInSe_2_/ZnS QDs becomes large for reduced re‐absorption loss. Figure 11b displays the optical image of CuInSe_2_/ZnS QD‐based LSC under visible light, showing dark color of such LSC that is caused by the low visible transmittance (VT). As shown in Figure 11c, the LSC's total sunlight absorptance (η_s,abs_) is 28% and related VT is only 23%. Figure 11d exhibits the LSC's IQE (denoted by hollow diamonds) with various device length of L.^97^ The EQE value of such CuInSe_2_/ZnS QD‐based LSC (12 cm × 12 cm) is ≈5.1%, showing ≈40% improvement than the highest value (3.7%) for similar CISeS‐QD based LSCs in literature.[[qv: 98a]]

Wei et al. fabricated a ternary eco‐friendly AgInS_2_/ZnS QD‐based LSC. The schematic diagram of such AgInS_2_/ZnS QD‐based LSC is shown in Figure 11e, which shows that the AgInS_2_/ZnS QDs are able to absorb the UV/visible light and emit the NIR photons. These AgInS_2_/ZnS QDs also show high PLQY of 60.3%. Figure 11g presents the photograph of AgInS_2_/ZnS QD‐based LSC with emitting concentrated light under UV illumination. Large Stokes shift (Figure 11f) was observed in this type of LSC, which is favorable for reducing the re‐absorption loss of QD‐based LSCs. As a result, the optical power efficiency of as‐fabricated AgInS_2_/ZnS QD‐based LSCs reached up to 3.94%.^76^

**Figure 12:**
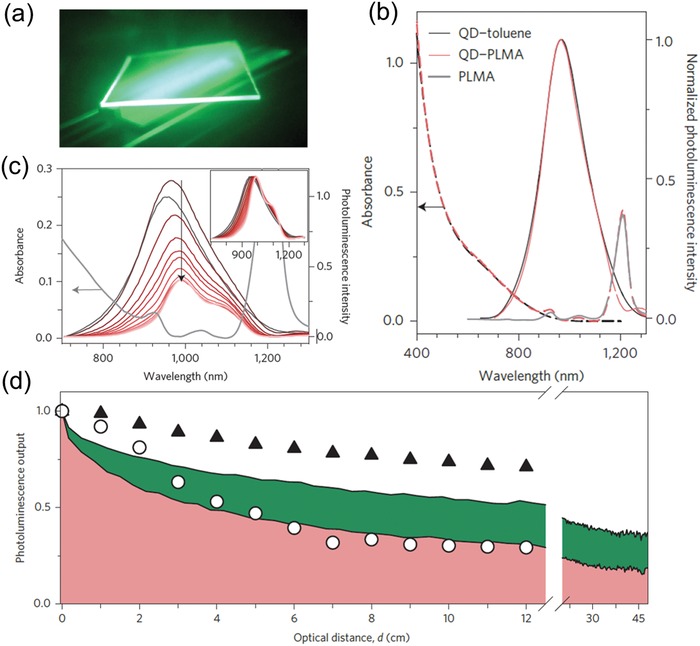
a) Optical image of CISeS /ZnS QD‐based LSC obtained from a UV‐filtered infrared camera. b) Absorption (dashed lines) and PL (solid lines) spectra of CISeS/ZnS QDs in solution (black) and in polymer matrix (pink). c) Absorption and PL spectra of the same LSCs in (a) excited at 532 nm. Inset image is normalized PL spectra of the as‐fabricated LSCs with varying *d*. d) The optical PL output with various optical distance in experimental and theoretical case. The circles represent the optical PL output featured by integrating the spectra in (c). The pink and green shadow represent the probability of a photon reaching the device edge, as simulated by Monte Carlo ray tracing. The triangles are the PL output provided by integrating the normalized PL spectra in the inset image of (c). Reproduced with permission.[[qv: 98a]] Copyright 2015, Springer Nature.

Meinardi et al. used the ternary alloyed “green” I–III–VI_2_ semiconductor of CuInSe_2_S_2−_ *_x_* (CISeS) QDs to fabricate LSC.[[qv: 98a]]] **Figure** **12**a shows the photograph of CISeS/ZnS QD‐based LSC obtained from a UV‐filtered infrared camera. Spectroscopic investigations of the polymer nanocomposites indicated the optical properties of CISeS/ZnS QDs are preserved even after the radical polymerization procedure. The absorption and PL spectra of the CISeS/ZnS QDs in the polymer matrix and toluene solution are similar, as shown Figure 12b. As depicted in Figure 12c, because of the scattering at optical imperfections within the polymer matrix and escaped photon from the waveguide, the non‐normalized PL intensity (main panel) decreased with increasing optical distance (*d*). Meanwhile, the shape of normalized PL spectra slightly changed with varying *d* (inset image), indicating that the re‐absorption losses of as‐fabricated LSC are very small.

Figure 12d presents the optical PL output with various optical distance in experimental and theoretical case. The circles represent the optical PL output featured by integrating the spectra in Figure 12c. The pink and green shadow represent the probability of a photon reaching the device edge, as simulated by Monte Carlo ray tracing. The triangles are the PL output provided by integrating the normalized PL spectra in the inset image of Figure 12c. Due to the synergistic effect of escaped light and re‐absorption by the QDs/polymer, the PL intensity is observed to decrease with increasing *d*. As‐fabricated CISeS/ZnS QD‐based LSC realized a high optical power efficiency of 3.2% due to the inhibited reabsorption and high PLQY of QDs.[[qv: 98a]]

### 4.4 Other Eco‐Friendly Nanocrystal‐Based LSCs

Other novel types of eco‐friendly nanocrystals have also emerged and been used to achieve high performance LSCs.^99^ Huang et al. fabricated an LSC based on eco‐friendly and aqueous‐solution‐processed gold nanoclusters.[[qv: 99b]] The glutathione‐stabilized gold nanoclusters (GSH‐AuNCs) are featured by ligand‐to‐metal charge‐transfer state and possess some fascinating optical properties for LSCs including large Stokes shifts, enhanced PL lifetime and nontoxicity.^100^ The absorption and emission spectra exhibit a very small spectral overlap owing to GSH‐AuNCs' special ligand‐to‐metal charge‐transfer (LMCT) state emission.[[qv: 99b]] As‐fabricated LSCs also show stable PL emission within the illumination time of 1500 s under an ambient environment.

**Figure 13:**
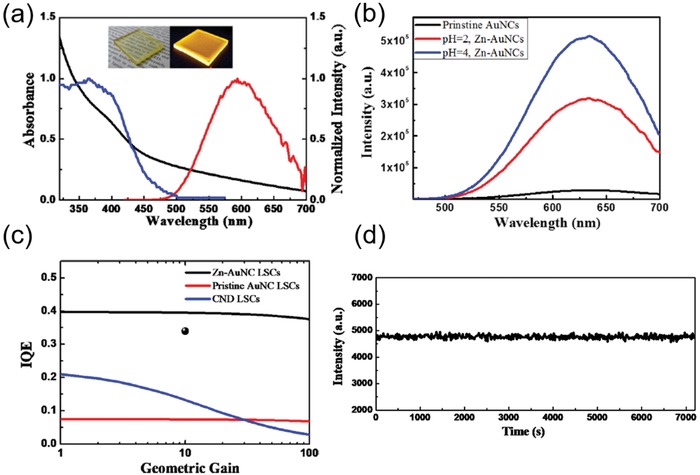
a) Absorption and emission spectrum for Zn‐AuNC‐based LSC. b) Emission spectrum for pristine AuNCs and Zn‐AuNCs at different pH values. c) Simulation of IQE of pristine AuNCs‐, CNDs‐, and Zn‐AuNC‐based LSC. d) Photostability test for Zn‐AuNC‐based LSCs under UV irradiation. Reproduced with permission.[[qv: 99a]] Copyright 2018, American Chemical Society.

Considering the poor PLQY of AuNCs, the same group developed a Zn^2+^‐induced cross‐linked AuNCs (Zn‐AuNCs) with enhanced PLQY.[[qv: 99a]] In this work, both Zn‐induced cross‐linked AuNCs in solution and incorporated in a solid matrix have been systematically studied. As‐fabricated Zn‐AuNC‐based LSCs showed improved PLQY of 53% and simultaneously possess a large Stokes shifts (**Figure** **13**a), which may be originated from the inhibition of nonradiative relaxation and the emissive states switching. With Zn^2+^ modification, the PL intensity of AuNCs dissolved in solution is increased from 0.4% to 7.7% at pH 4, as shown in Figure 13b. Notably, these Zn‐AuNC‐based LSCs exhibited a high IQE of 34–36%.[[qv: 99a]] They also compared the IQE as a function of geometric gain G among Zn‐AuNCs‐, pristine AuNCs‐, and N‐doped carbon QD‐based LSCs, as exhibited in Figure 13c. It was found that the IQE of CND‐based LSCs dropped apparently with the increasing G due to its low PLQY and rather large re‐absorption loss. In contrast, Zn‐AuNCs‐ and pristine AuNC‐based LSCs maintained its IQE with increasing G due to their small Stokes shift. Owing to largely enhanced PLQY of Zn‐AuNCs, as‐fabricated LSCs maintained its highest IQE about 0.37 with G increased to 100, which can be regarded as efficient eco‐friendly LSCs as compared to other LSCs comprised of toxic QDs. Furthermore, the photostability of Zn‐AuNC‐based LSCs under irradiation was displayed in Figure 13d, in which the PL emission can maintain its original intensity for 2 h at an ambient condition.

In order to provide a comprehensive understanding of these various kinds of eco‐friendly nanomaterial‐based LSCs, the parameters of the eco‐friendly CQD‐based and other nanomaterial‐based LSCs including absorption and PL emission range, PLQY, Stokes shift, LSC's size, optical efficiency, and PCE are shown in **Table** **1**.

**Table 1:** Comparison of the parameters of various eco‐friendly CQD/nanocrystal‐based LSCs

Fluorophore	QY [%]	Absorption range [nm]	Emission range [nm]	Stokes shift	LSC size [cm^3^]	Optical efficiency [%]	PCE [%]	Refs
N‐doped carbon QDs	–	300–550	400–650	–	2.5 × 1.6 × 0.1	4.75	3.94	[[qv: 18a]]
N‐doped carbon QDs	–	UV to 420	400–500	–	1.8 × 1.8 × 0.11	5.02	4.97	88
Carbon QDsa)	40	UV to 550	450–600	–	10 × 10 × 0.2	1.1	–	[[qv: 18b]]
Si QDs	55	UV to 600	600–1000	400 meV	12 × 12 × 0.26	2.85	–	[[qv: 18g]]
InP/ZnO QDs	–	UV to 550	550–700	–	9 × 1.5 × 0.3	1.45	–	[[qv: 12d]]
CuInS_2_/ZnS QDs	91	UV to 830	620–1240	> 550 meV	10 × 10 × –	8.1	2.94	[[qv: 20a]]
CuInS_2_/ZnS QDs	56	UV to 500	450–750	> 150 nm	2.2 × 2.2 × 0.3	26.5	8.71	95
CuInS_2_/ZnS QDs	65	UV to 500	450–700	–	2 × 2 × 0.8	–	4.20	[[qv: 18f]]
Zn and Al co‐doped CuInS_2_ QDs	–	UV to 800	600–900	–	1.8 × 1.8 × 0.11	6.97	3.18	96
CuInSe_2_/ZnS QDsa)	72	UV to 800	650–1000	–	15.2 × 15.2 × 0.16	6.4	3.1	97
CuInSe_2_S_2−_ *_x_* QDs	40	UV to 900	800–1250	530 meV	12 × 12 × 0.3	3.2	–	[[qv: 98a]]
AgInS_2_/ZnS QDs	60	UV to 800	500–1240	–	5 × 5 × 3	3.94	–	76
GSH‐AuNCs	–	UV to 500	500–800	–	2 × 2 × 0.2	–	–	[[qv: 99b]]
Zn‐AuNCs	53	UV to 500	350–550	–	2 × 2 × 0.2	–	–	[[qv: 99a]]

^a)^ Tandem LSC.

## 5 Conclusion and Perspectives

In conclusion, this review summarized latest developments of state‐of‐the‐art LSCs based on various kinds of unary, binary and ternary eco‐friendly QDs. The most promising feature of these eco‐friendly QDs is their low‐toxicity as compared to current widely used Cd and Pb‐based QDs, which enables the fabrication of environmental‐friendly QD‐based optoelectronic devices (e.g., LSCs) that are not harmful to human health and natural environment. An overview of synthetic approaches and surface engineering of several typical unary/binary/ternary eco‐friendly QDs, including carbon QDs, Si QDs, InP QDs, CuInS_2_ QDs, CuInSe_2_ QDs, AgInS_2_ QDs, etc., have been provided and discussed. These modification techniques can effectively optimize and tune the optical properties of QDs, leading to excellent PLQY, large Stokes shift and high photostability. The performance of as‐fabricated LSCs based on these optimized eco‐friendly QDs and other nanomaterials (e.g., AuNCs) have been analyzed and highlighted as well. Despite the remarkable effort devoted to the synthesis and optical properties of environmental‐friendly QDs and relevant LSCs application, there are still several critical limitations and challenges to overcome in eco‐friendly QD‐based LSCs:

i) Most of current widely used synthetic methods of eco‐friendly QDs require high temperature and the yield of QDs is relatively low. Hence, low‐temperature and large‐scale synthetic techniques of eco‐friendly QDs should be exploited to reduce the overall energy consumption and improve the productivity during QDs' synthesis.
ii) Although these environmental‐friendly QDs are comprised of nontoxic elements, the organic precursors involved in the synthesis are still hazardous. It is highly desired to explore nontoxic organic precursors and achieve absolutely “green” merit of QDs along the whole synthesis processes.
iii) The optical properties of eco‐friendly QDs still need to be further optimized. Additional effective approaches toward QDs' modifications including surface engineering (e.g., core/shell/shell structure, defects passivation/modulation, etc.) and co‐doping method should be developed, which is favorable to obtain QDs with higher PLQY, larger Stoke‐shift and long‐term photostability.
iv) The efficiency of these eco‐friendly QD‐based LSCs is still very low and hard to realize further device commercialization. Despite the optimization of QDs in LSCs, some new architectures of eco‐friendly LSCs should be designed to promote their practical application in future urban buildings, for instance, the design of tandem LSCs that effectively enhance the absorption efficiency and optical conversion efficiency.
v) The size of current eco‐friendly QD‐based LSCs is still very small and should be scaled up while simultaneously maintaining their high efficiency. Besides, reducing the cost of LSCs fabrication/installation is encouraged as well.

In brief, eco‐friendly QD‐based LSCs still have much space for improvements. With further optimizations, high efficiency, large‐scale, and stable eco‐friendly QD‐based LSCs can be achieved to realize net‐zero power consumption of future urban buildings.

## Conflict of Interest

The authors declare no conflict of interest.
